# Provision of community-based mental health care, Latvia

**DOI:** 10.2471/BLT.19.239913

**Published:** 2020-04-21

**Authors:** Maris Taube, Wilm Quentin

**Affiliations:** a Riga Stradiņš University, Riga Centre of Psychiatry and Narcology, Veldres 1a, Riga, LV 1064, Latvia.; b European Observatory on Health System and Policies, Department of Health Care Management, Technische Universität Berlin, Berlin, Germany.

## Abstract

**Problem:**

In Latvia, the move towards community-based mental health-care services has been slow.

**Approach:**

The hospital managers of the only psychiatric hospital in Riga decided to establish two community-based clinics that were financially and administratively integrated with the hospital. The clinics were established using a step-wise approach by redistributing resources, including psychiatrists, nurses and beds, from the hospital to the new clinics. In 2005, the Veldre clinic started outpatient consultations and day care admissions. In 2009, Pardaugava clinic opened as an outpatient clinic. In 2012, an open-door inpatient ward with 30 beds was transferred from the psychiatric hospital to Veldre. In 2013, Pardaugava clinic opened a day care clinic and an open-door inpatient ward, transferring 26 beds from the psychiatric hospital.

**Local setting:**

Latvians have worse mental health indicators than those of the average population in Europe. Mental care has traditionally focused on inpatient care.

**Relevant changes:**

The clinics are now providing most of the outpatient services and the number of inpatients treated at the hospital has declined from 5696 patients in 2004 to 4957 patients in 2018. Patients are treated in a more open and patient-centred environment.

**Lessons learnt:**

The administrative and financial integration of the new community-based clinics within the existing structures of the hospital is a successful approach. Transferring resources to the clinics seems to have improved the quality of care without requiring additional funding apart from the initial investment costs. Involving the staff members during the planning phase reduced resistance to the project.

## Résumé

**Problème:**

En Lettonie, l'évolution vers des prestations de soins de santé mentale communautaires a progressé lentement.

**Approche:**

Les dirigeants de l'unique hôpital psychiatrique de Riga ont décidé de fonder deux cliniques communautaires intégrées financièrement et administrativement à l'hôpital. Leur création a fait l'objet d'une approche graduelle de redistribution des ressources, notamment des psychiatres, infirmiers et lits, depuis l'hôpital vers les nouvelles cliniques. En 2005, la clinique de Veldre a commencé les consultations externes et les admissions pour des soins de jour. En 2009, la clinique externe de Pardaugava a débuté ses activités. En 2012, une unité d'hospitalisation à régime ouvert comptant 30 lits a été transférée de l'hôpital psychiatrique vers Veldre. En 2013, la clinique de Pardaugava a inauguré un établissement de jour et une unité d'hospitalisation à régime ouvert, récupérant 26 lits de l'hôpital psychiatrique.

**Environnement local:**

Les indicateurs de santé mentale en Lettonie sont inférieurs à la moyenne observée au sein de la population européenne. Les soins de santé mentale étaient traditionnellement axés sur l'hospitalisation des patients.

**Changements significatifs:**

Les cliniques assurent désormais la plupart des prestations externes et le nombre d'hospitalisations a diminué, passant de 5696 patients en 2004 à 4957 patients en 2018. Les patients sont pris en charge dans un environnement plus ouvert et mieux adapté à leurs besoins.

**Leçons tirées:**

L'intégration administrative et financière des nouvelles cliniques communautaires au sein des structures hospitalières existantes est une approche efficace. Le transfert des ressources vers les cliniques semble avoir amélioré la qualité des soins prodigués sans nécessiter de fonds supplémentaires, à l'exception de l'investissement initial. Enfin, impliquer les membres du personnel dans la phase de planification a permis de limiter la résistance au projet.

## Resumen

**Situación:**

El avance hacia los servicios de atención de la salud mental comunitarios es muy lento en Letonia.

**Enfoque:**

Los gerentes del único hospital psiquiátrico de Riga decidieron establecer dos clínicas en la comunidad que se integraron financiera y administrativamente con el hospital. Las clínicas se establecieron mediante un enfoque gradual, al redistribuir los recursos, incluidos psiquiatras, enfermeros y camas, del hospital a las nuevas clínicas. En 2005, la clínica de Veldre inició consultas a pacientes ambulatorios y admisiones para la atención diurna. En 2009, la clínica Pardaugava se inauguró como una clínica para pacientes ambulatorios. En 2012, una sala para pacientes hospitalizados con 30 camas fue transferida del hospital psiquiátrico a Veldre. En 2013, la clínica Pardaugava abrió una clínica de atención diurna y una sala para pacientes hospitalizados sin restricciones, en la que se transfirieron 26 camas del hospital psiquiátrico.

**Marco regional:**

Los habitantes de Letonia tienen indicadores de salud mental peores que los de la población media de Europa. Tradicionalmente, la atención mental se ha centrado en la atención a los pacientes hospitalizados.

**Cambios importantes:**

En la actualidad, las clínicas prestan la mayor parte de los servicios a los pacientes ambulatorios y el número de pacientes hospitalizados tratados en el hospital disminuyó de 5696 pacientes en 2004 a 4957 en 2018. Los pacientes son tratados en un ambiente más accesible y centrado en el paciente.

**Lecciones aprendidas:**

La integración administrativa y financiera de las clínicas nuevas en la comunidad dentro de las estructuras que ya existen en el hospital es un enfoque exitoso. La transferencia de recursos a las clínicas parece haber mejorado la calidad de la atención sin requerir el financiamiento adicional aparte de los costos de inversión inicial. La participación de los miembros del personal durante la fase de planificación redujo la complejidad del proyecto.

## ملخص

المشكلة كان التحرك نحو خدمات الرعاية الصحية النفسية المجتمعية بطيئاً في لاتفيا.

الأسلوب قرر مديرو المستشفى النفسي الوحيد في ريجا إنشاء عيادتين مجتمعيتين، تم دمجهما مالياً وإدارياً مع المستشفى. تم إنشاء العيادات باستخدام نهج متدرج الخطوات عن طريق إعادة توزيع الموارد، بما في ذلك الأطباء النفسيين والممرضات والأسرّة، من المستشفى إلى العيادات الجديدة. وبدأت عيادة فيلدري في عام 2005 تقديم خدمة استشارات العيادات الخارجية وقبول مرضى الرعاية النهارية. وتم في عام 2009 افتتاح عيادة بارداوجافا كعيادة خارجية. وتم في عام 2012 نقل جناح مفتوح للمرضى الداخليين بطاقة 30 سريراً، من مستشفى الطب النفسي إلى فيلدري. في عام 2013، تم افتتاح عيادة بارداوجافا للرعاية النهارية، وجناح مفتوح للمرضى الداخليين، مع نقل 26 سريرًا من مستشفى الطب النفسي.

المواقع المحلية يعاني سكان لاتفيا من مؤشرات للصحة النفسية أسوأ من المستوى المتوسط في أوروبا. تركز الرعاية النفسية بشكل تقليدي على رعاية المرضى الداخليين.

التغيّرات ذات الصلة تقدم العيادات الآن معظم خدمات المرضى الخارجيين، كما انخفض عدد المرضى الداخليين الذين تم علاجهم في المستشفى من 5696 مريضاً في عام 2004، إلى 4957 مريضاً في عام 2018. ويتم علاج المرضى في بيئة أكثر انفتاحاً وتركيزاً على المريض.

الدروس المستفادة يعتبر التكامل الإداري والمالي للعيادات المجتمعية الجديدة ضمن الهياكل الحالية للمستشفى، أسلوباً ناجحاً. ويبدو أن نقل الموارد إلى العيادات قد أدى إلى تحسين جودة الرعاية دون الحاجة إلى تمويل إضافي بغض النظر عن تكاليف الاستثمار الأولية. لقد أدى إشراك أعضاء فريق العمل أثناء مرحلة التخطيط إلى الحد من مقاومة المشروع.

## 摘要

**问题:**

拉脱维亚当前以社区为基础的心理健康护理服务进展缓慢。

**方法:**

里加唯一一家精神病医院的管理人员决定建立两个以社区为基础的诊所，这两家诊所的财务与行政事项纳入医院系统中由医院统一管理。诊所采用逐步实施的方法建立，将医院中的精神科医生、护士和床位等资源重新分配给新诊所。2005 年，Veldre 诊所开始接受门诊咨询和日间入院护理。2009 年，开设 Pardaugava 诊所，该诊所为门诊诊所。2012 年，一间有 30 张床位的开放式住院病房从精神病院转移到了 Veldre。2013 年，Pardaugava 诊所开设了一家日间护理诊所和一间开放式住院病房，从精神病医院转移了 26 张床位。

**当地状况:**

拉脱维亚人口心理健康指标低于欧洲人口平均指标。心理健康护理还是偏传统方式，更侧重于住院护理。

**相关变化:**

这些诊所现在提供大部分的门诊服务，在医院接受治疗的住院患者从 2004 年的 5696 人下降到 2018 年的 4957 人。患者在更加开放和以患者为中心的环境中接受治疗。

**经验教训:**

将这些新社区诊所的财务与行政事项纳入医院现有结构系统范围内是一种成功的做法。除了初始投资成本外，将资源转移到诊所似乎改善了护理质量，且不需要额外的资金。让工作人员参与规划阶段可以减少项目阻力。

## Резюме

**Проблема:**

В Латвии осуществляется медленный переход к оказанию психиатрической помощи на уровне общин.

**Подход:**

По решению руководства единственной психиатрической больницы в Риге было создано две клиники на базе общин, которые являлись частью больницы в административном и финансовом плане. Клиники создавались с использованием поэтапного подхода к перераспределению ресурсов, включая психиатров, медсестер и койко-места, из больницы в новые клиники. В 2005 году клиника «Вельдре» начала проводить амбулаторные консультации и принимать пациентов в дневном стационаре. В 2009 году была открыта амбулаторная клиника в Пардаугаве. В 2012 году стационарное отделение свободного режима на 30 койко-мест было переведено из психиатрической больницы «Вельдре». В 2013 году клиника в Пардаугаве открыла дневной стационар и стационарное отделение свободного режима, приняв 26 койко-мест из центральной психиатрической больницы.

**Местные условия:**

У жителей Латвии показатели психического здоровья хуже, чем в среднем для населения Европы. Психиатрическая помощь традиционно осуществлялась в основном в стационаре.

**Осуществленные перемены:**

В настоящее время клиники предоставляют большинство амбулаторных услуг, и количество пациентов в стационаре больницы снизилось с 5696 человек в 2004 году до 4957 в 2018 году. Пациенты получают лечение в более свободной и ориентированной на их потребности атмосфере.

**Выводы:**

Административная и финансовая интеграция новых клиник на базе общин в существующую инфраструктуру больницы оказалась успешной. Перенос ресурсов в клиники способствовал улучшению качества обслуживания без привлечения дополнительного финансирования сверх первоначальных инвестиций. Вовлечение персонала на этапе планирования позволило сократить сопротивление реализации данного проекта.

## Introduction

The World Health Organization (WHO) promotes the development of community-based mental health-care services as part of a balanced care model.^1^^,^^2^ Nevertheless, policy-makers in many central and eastern European countries have struggled to develop adequate community mental health services, despite having national policies for such care provision in place.^1^^,^^3^

Here we present the approach for establishing two community-based mental health clinics in Latvia.

## Local setting

Latvia is a Baltic country with about 2 million inhabitants. Mental health indicators in Latvia are worse than that of other European populations. For example, the number of deaths per 100 000 people caused by suicide or intentional self-harm was 16.2 in 2015, compared with 9.8 overall in the WHO European Region.^4^ Mental care has traditionally focused on inpatient care, and the move towards community-based mental health services has often taken place in response to pressures from international and human rights organizations.^5^ For example, WHO and the health ministry found in 2005 an insufficient development of services outside of psychiatric hospitals.^6^ Although the number of psychiatric hospital beds has decreased from 186 beds per 100 000 population in 2000, to 126 beds per 100 000 population in 2014, the number is higher than the European average of 68 beds per 100 000 population.^4^ In the capital Riga, with about 641 000 inhabitants, one psychiatric hospital, the Riga Psychiatry and Narcology Centre, provided mental care.^4^

## Approach

To tailor service provision to patient needs and to enable greater continuity of care across outpatient and inpatient settings, the psychiatric hospital directors and chairman of the board decided in 2002 to establish a community-based clinic in Riga. Based on hospital statistics, the clinic should provide care for 4000 to 5000 patients living nearby the clinic, and therefore the location needed to be sufficiently populated. The location should also be accessible by public transport and be in a different administrative district than the hospital. 

In 2005, the Veldre clinic started outpatient consultations and day care admissions after the hospital renovated an unused kindergarten building. To promote community integration and reduction of stigma, the clinic was located next to a school. Based on the example of the Veldre clinic, a second clinic, Pardaugava, opened in 2009 as an outpatient clinic after renovating a rehabilitation facility for substance abusers and constructing a new building. In 2012, an open-door inpatient ward with 30 beds was transferred from the psychiatric hospital to Veldre. Finally, in 2013, Pardaugava opened a day care clinic and an open-door inpatient ward, transferring 26 beds from the locked ward at the psychiatric hospital.

To set up the clinics, the hospital managers decided to redistribute resources, including psychiatrists, nurses and beds, from the hospital to the clinics. All relevant staff members were involved in the planning of the new clinics.

Hospital staff members would keep their jobs but would have to work at the new locations, full- or part-time. Eventually, the clinics employed more staff for outpatient care since the patient load increased. Part-time staff usually work at the psychiatric hospital for the rest of the week. To ensure continuity, an outpatient requiring inpatient care is treated by the same psychiatrist. 

As special training courses in community-based mental health care were unavailable, most community-based practice skills had to be acquired on the job. However, continuing education activities were available. For example, six times a year the Latvian Psychiatric Association Conferences provided free lectures lasting 3–4 hours, covering topics such as psychiatric rehabilitation, multidisciplinary team work and role of art therapies. Furthermore, three to five senior staff had the opportunity to visit similar facilities in Finland and the United Kingdom of Great Britain and Northern Ireland.

Patients receive psychiatric outpatient consultations without a referral, from 8:00 to 18:00, and patients with more complex needs are admitted for day care or inpatient care at the clinics. Admitted patients receive a tailored rehabilitation and treatment programme, including cognitive behavioural therapy, pharmacotherapy, art therapy, ergotherapy and physiotherapy. The development of the programme was informed by the literature and programmes in other countries. The day care programme focuses on nonpharmacological treatment, while inpatient care, which is reserved for patients with highly complex needs, focuses on, for example, identifying medications for treatment. Treatment is free of charge.

The psychiatric hospital is legally and financially responsible for the clinics and the directors of the clinics report to the chairman of the hospital board. Strong incentives exist at the institutional level to develop outpatient and day care activities. The inpatient care is financed based on a global budget, and funding remains the same over time, while budgets for outpatient and day care are determined based on registered fee-for-service points. With increasing activity, the increasing outpatient and day care budget allowed to finance new positions. Inpatient service provision at the clinics did not require additional funding apart from the initial investments for construction and equipment of the new facilities, as the inpatient care budget was simply transferred from the hospital to the clinics. The total investment costs were 4.4 million euros (€), of which € 3.7 million was financial support from the European Union. Incentives for outpatient care provision exist also at the individual level as psychiatrists (since 2005) and therapists (since 2019) receive an additional €7 fee-for-service based remuneration for outpatient visits from the health insurance system. 

## Relevant changes

In 2019, each clinic had seven psychiatrists employed on full-time contracts and four to seven psychiatrists working six hours per week. Between 2005 and 2013, 43 staff members were transferred from the hospital to the clinics. In addition, the clinics have hired 12 psychiatrists, eight nurses and 27 therapeutic specialists, either full time or part-time.

The clinics are now providing most of the outpatient services. In 2018, only 16 310 (29%) of the 55 997 visits took place in the hospital. Of the remaining outpatient visits, the Veldre clinic had 18 979 visits and Pardaugava clinic had 20 708 visits. The number of inpatients treated at the hospital has declined from 5696 patients in 2004 to 4957 patients in 2018. During the same period, the total number of inpatient days decreased from 318 541 days to 164 084 days. In 2018, 15% (886/5843) of inpatients were treated at the clinics. The number of patients receiving day care treatment increased from 218 in 2004 to 457 in 2018.

The types of disorders treated by inpatient care differ between the clinics and the hospital. At the hospital such care is focused on psychosis and other mental disorders, while the clinics attract mostly patients with affective disorders, including depression, anxiety and bipolar disorders (Table 1).

**Table 1:** Distribution of mental health diagnoses at the hospital and community-based clinics, Latvia, 2019

Diagnosis (ICD-10 code)^a^	No. of patients (%)							
	Hospital^b^			Veldre clinic			Pardaugava clinic	
	Inpatient *n* = 4901	Outpatient *n* = 5719		Inpatient *n* = 425	Outpatient *n* = 6061		Inpatient *n* = 399	Outpatient *n* = 6568
Organic, including symptomatic, mental disorders (F0)	1370 (28.0)	1736 (30.4)		60 (14.1)	1869 (30.8)		57 (14.3)	1829 (27.8)
Schizophrenia, schizotypal and delusional disorders (F2)	2345 (47.8)	2258 (39.5)		140 (32.9)	2234 (36.9)		169 (42.4)	2126 (32.4)
Mood [affective] disorders (F3)	634 (12.9)	715 (12.5)		185 (43.5)	923 (15.2)		159 (39.8)	1091 (16.6)
Neurotic, stress-related and somatoform disorders (F4)	346 (7.1)	686 (12.0)		38 (8.9)	717 (11.8)		13 (3.3)	1094 (16.6)
Behavioural syndromes associated with physiological disturbances and physical factors (F5)	NA	38 (0.7)		NA	42 (0.7)		NA	58 (0.9)
Disorders of adult personality and behaviour (F6)	51 (1.0)	60 (1.0)		2 (0.5)	24 (0.4)		1 (0.3)	65 (1.0)
Mental retardation (F7)	134 (2.7)	226 (4.0)		0 (0.0)	252 (4.2)		0 (0.0)	305 (4.6)
Behavioural and emotional disorders with onset usually occurring in childhood and adolescence (F8)	21 (0.4)	0 (0.0)		0 (0.0)	0 (0.0)		0 (0.0)	0 (0.0)

NA: not applicable; ICD: International Classification of Diseases.

^a^ The categories are based on the main diagnostic groups of *International Statistical Classification of Diseases and Related Health Problems*, 10th edition.^7^

^b^ The Riga Psychiatry and Narcology centre is the only psychiatric hospital in Riga.

Note: Inconsistencies may arise due to rounding.

Source: Riga Psychiatry and Narcology centre.

Limited data are available on the quality of mental care in Latvia and quality has not been systematically monitored.^8^ However, psychiatrists in the clinics receive positive feedback from patients on the continuity of care. Furthermore, the care provided at the clinics is much more in line with international recommendations for a balanced care approach.^1^ In addition, the clinics have piloted an adapted version of the Psychiatric Inpatient Patient Experience Questionnaire^8^ and the results showed that patients were generally satisfied with the care they received in the clinics.^9^

## Lessons learnt

The two clinics have been able to implement the community-based balanced care approach, treating patients in a more open and patient-centred environment. Patients and their psychiatrists appreciate this approach and personal experience of treating psychiatrists suggests that managing patients in a day-care setting reduces hospitalizations. However, demonstrating the effect of less hospitalization is difficult due to limited data availability.

Personal motivation of the hospital managers at the psychiatric hospital played an important role in the development of the clinics. Hospital managers wanted to be progressive and replace inpatient care with day care and outpatient care. Furthermore, health-care financing principles in Latvia stimulate the development of outpatient and day care activity.^10^

The idea of establishing community-based clinics initially met resistance from psychiatrists and nurses. Staff members were reluctant to adopt new practices as these required a different mindset and new skills, for example, suicide risk management and dealing with patients not attending day-care treatment. In addition, many staff members were reluctant to relocate because of longer commuting time.

However, hospital managers addressed staff resistance by involving staff members in the planning process, by incorporating their suggestions in designing and equipping the new facilities, and by allowing them to shape the organization of work. Some staff were easier to convince of the benefits of community-based practice. Financial incentives for the provision of outpatient care also helped. In addition, some psychiatrists and nurses already lived close to the new clinics. In 2007, the director of Pardaugava clinic was elected President of the Latvian Psychiatric Association, which contributed to acceptance of the clinics.

The Latvian experience holds several lessons for other countries wishing to move psychiatric service provision away from hospitals and towards community-based care (Box 1). First, external pressure and recommendations of international organizations, as well as external funding can contribute to developing community-based mental health services.^11^ Second, the administrative and financial integration of the new clinics within the existing structures of the hospital and the involvement of clinical staff in the planning process created a sense of security for the involved personnel and encouraged them to support the initiative. Third, the approach of transferring resources to a community-based clinic seemed to improve the quality of care without requiring additional funding apart from the initial investment costs. Finally, our experience shows that incentives of the financing system are important. The hard budget constraints for inpatient care combined with activity-based funding for outpatient and day care, coupled with fee-for-service-based remuneration for treating specialists created incentives that promoted the establishment of the clinics and the expansion of activity.

Box 1 **Summary of main lessons learnt**

- External pressure by international organizations stimulated national efforts to develop community-based psychiatric care.
- Financial incentives to support the development of outpatient and day-care settings played an important role.
- Transferring resources from the hospital to the community-based clinics improved the quality of care without requiring additional funding.

In 2019, the government adopted the Plan of Improving Access to Mental Health Care 2019–2020.^10^ The plan suggests the creation of seven new community-based mental health clinics across the country, modelled after the clinics in Riga. The two existing clinics will provide in-service education for staff of the new clinics, and the Latvian version of the quality questionnaire will be used in other mental health institutions. To strengthen the community outreach in Riga, plans exist to enable nurses to do home visits and family therapies.
